# Effect of shunt-dependency on long-term outcome after aneurysmal subarachnoid hemorrhage: a post-hoc analysis of the EARLYDRAIN prospective patient cohort

**DOI:** 10.1007/s10143-025-03510-4

**Published:** 2025-04-15

**Authors:** Vesna Malinova, Dorothee Mielke, Veit Rohde, Peter Vajkoczy, Stefan Wolf

**Affiliations:** 1 Department of Neurosurgery, University Medical Center Göttingen, Göttingen, Germany; 2 Department of Neurosurgery, Carité Universitätsmedizin Berlin, Berlin, Germany; 3 Department of Neurosurgery, University Hospital Augsburg, Augsburg, Germany; 4 Department of Neurosurgery, Georg-August-University, Robert-Koch-Straße 40, 37075 Göttingen, Germany

**Keywords:** Subarachnoid hemorrhage, Posthemorrhagic hydrocephalus, Shunt-dependency

## Abstract

Shunt-dependent hydrocephalus is common after aneurysmal subarachnoid hemorrhage (aSAH) and has the risk to compromise the functional recovery of affected patients. This study investigated whether shunt-dependency is associated with the long-term outcome after aSAH. A post-hoc analysis was performed using the patient cohort of a prospective randomized controlled trial (EARLYDRAIN) conducted between 2011 and 2016. Patients were randomized for an early lumbar drainage (144 patients) or standard treatment alone (143 patients). Shunt insertion within 180 days after ictus was considered as shunt-dependency. The modified Rankin scale (mRS) at 180 days (mRS ≤ 2 equaling favorable outcome) was used for outcome assessment. The study population involved 287 aSAH patients with a mean age of 55 years. Shunt-dependent hydrocephalus was found in 29% of all patients. Patients without shunt-dependency had a better functional outcome at discharge (mRS 3.2 ± 2.0) compared to shunt-dependent patients (mRS 3.9 ± 1.5). Univariate analyses revealed worse functional outcome at 180 days in the patients with shunt-dependency compared to those without shung-dependency (mRS 2.8 ± 1.7, vs. mRS 2.2 ± 2.3). In multivariate analysis, adjusted for age, severity of the initial subarachnoid hemorrhage and the use of a lumbar drain, shunt-dependency was not associated with long-term outcome (*p* = 0.26). After correction for age, treatment and SAH-grade, shunt-dependency did not show an association with the outcome in aSAH patients after rehabilitation. Clinical improvement due to rehabilitation after shunt insertion may be the explanation why chronic hydrocephalus per se does not represent an independent factor associated with long-term outcome.

## Introduction

Hydrocephalus is a common complication of aneurysmal subarachnoid hemorrhage (aSAH) usually starting as an acute disturbance of cerebrospinal fluid (CSF) circulation affecting up to 70% of patients and resulting into a chronic hydrocephalus requiring permanent CSF diversion in 30% of cases [1–3]. Shunt-dependent hydrocephalus is associated with neurological impairment that has been previously shown to correlate with poor outcome after aSAH [4–6]. Recognizing and addressing modifiable risk factors for shunt-dependency is clinically relevant to reduce the necessity of permanent shunts in aSAH patients [7–8]. The pathophysiology of shunt-dependent hydrocephalus involves multiple pathways including inflammation, oxidative injury, apoptosis, neuronal death, and fibrosis of the CSF tract [3]. The amount of intracranial blood and blood degradation products play an important role in these processes contributing not only to early brain injury and delayed cerebral ischemia (DCI) but also to chronic hydrocephalus after aSAH [9]. An early CSF diversion via external ventricular drain (EVD) or lumbar drain (LD) is applied to treat acute hydrocephalus and reduce intracranial pressure, which can also contribute to blood clearance after aSAH [10]. In the recently published EARLYDRAIN trial, the impact of early LD on delayed infarctions and functional outcome 6 months after aSAH was evaluated. Main finding was a reduced incidence of infarctions and a decreased rate of unfavorable outcome at 6-months follow-up [11]. Interestingly, there was no difference in the rate of shunt-dependency between patients with and those without early LD in the EARLYDRAIN trial [11]. This finding arose the question, whether shunt-dependency per se represents an independent predictor of functional outcome after aSAH, which was the rationale for conducting this post-hoc analysis of the EARLYDRAIN data. This study investigated the association of shunt-dependency with functional outcome at 180 days after aSAH.

## Methods

### Trial design

In this study, a post-hoc analysis was performed using the prospectively collected data of the patient cohort included in the EARLYDRAIN trial. EARLYDRAIN was a multicenter randomized controlled trial with a 1:1 ratio randomization to receive either standard of care or the additional use of a lumbar drain. Ethics approval was provided by the Lead Ethics Committee of the University of Erlangen, Germany, and corresponding boards at each affiliated center, as required. The study was conducted according to the Declaration of Helsinki.

### Participants

The EARLYDRAIN collective consisted of 287 randomized patients analyzed according to the intention-to-treat principle with 144 receiving an early LD additional to standard of care and 143 patients receiving only standard of care. Main inclusion criteria were patients with aSAH older than 18 years with aneurysm treatment performed within 48 h after ictus. The additional placement of an EVD was allowed and left at the discretion of the treating neurosurgeons at the participating center. Consent to aneurysm treatment and study inclusion was provided by the patients in case of capability, or their legally authorized representatives.

### Data evaluated

The EARLYDRAIN case report file recorded the dates of subarachnoid hemorrhage, aneurysm treatment, eventual shunt surgery, discharge from the acute hospital and long-term outcome assessment. Clinical data included treatment features and complications and assessment of vasospasm. The diagnosis of clinical vasospasm was at the judgment of local investigators, TCD vasospasm was determined by mean flow thresholds of 120 cm/s and 160 cm/s in the middle cerebral artery, and angiographic vasospasm by the highest degree of vessel narrowing on assessment, compared to the baseline image after the initial hemorrhage.

Timing and indication of shunt placement was at the discretion of local investigators. Treatment with a lumbar drain was considered as actually treated in the EARLYDRAIN trial, reflecting at least 4 days of 120 ml daily lumbar drainage within the first 8 days after aneurysm occlusion.

### Outcome assessment

Neurological outcome assessment was performed with the modified Rankin Score (mRS) by clinical personnel not involved in the main study. The primary outcome in this post-hoc analysis was the shunt-dependency at 180 days follow-up and its association with the functional outcome. A mRS ≤ 2 was regarded as a favorable outcome.

### Statistical analysis

The statistical analyses were performed by means of the GraphPad Prism software (Version 9, GraphPad Software, San Diego, CA, USA) and R (R 4.1.1, R Foundation for Scientific Computing, Vienna, Austria). For the presentation of baseline data descriptive statistics and frequency distribution analysis was performed. Continuous variables are depicted as mean ± standard deviation (SD), categorical variables as frequency or percentages. Descriptive statistics was used for calculation of baseline characteristics in the study population. Univariate and multivariate regression analyses were performed with generalized linear regression models to identify factors associated with shunt dependency and functional outcome. Factors with *p* < 0.1 in univariate analysis were considered as potentially relevant for shunt dependency. The main EARLYDRAIN manuscript did already identify age, clinical hemorrhage severity, intraparenchymal and intraventricular hemorrhage as well as the use of a lumbar drain as related to neurologic outcome after 6 months. Shunt placement at any time was then investigated as further co-factor. For regression analysis, functional outcome was dichotomized at mRS ≤ 2 vs. mRS ≥ 3. Differences were considered statistically significant at *p* < 0.05. We regard our analysis as explorative and therefore did not apply corrections for multiple testing.

## Results

### Patient characteristics

A total of 287 patients was included in the analysis of this study. The mean age in the study population was 55.5 ± 12.4 years. A high WFNS grade IV or V was found in 46%, and 92% of all patients had a high modified Fisher grade 3 or 4. The baseline characteristics of the study population are summarized in Table 1. The treatment characteristics are shown in Table 2.

**Table 1:** Baseline characteristics under consideration of shunt-dependency

Characteristic	VP shunt	No shunt required	*p* value
	*n* = 83	*n* = 204	
Mean age in years (range)	59 (52 to 68)	52 (46 to 61)	< 0.001
Sex n (%)			
- Female - Male	57 (68.7) 26 (31.3)	140 (68.6) 64 (31.4)	1
mRS on admission n (%)			
- 0 - 1	81 (97.6) 2 (2.4)	188 (92.2) 16 (7.8)	0.15
Hunt & Hess n (%)			
- 1 - 2 - 3 - 4 - 5	11 (13.3) 16 (19.3) 13 (15.7) 17 (20.5) 26 (31.3)	43 (21.1) 53 (26) 46 (22.5) 27 (13.2) 35 (17.2)	0.02
WFNS grading n (%)			
- 1 - 2 - 3 - 4 - 5	16 (19.3) 14 (16.9) 2 (2.4) 8 (9.6) 43 (51.8)	79 (38.7) 29 (14.2) 15 (7.4) 21 (10.3) 60 (29.4)	0.001
Modified Fisher grading n (%) - 1 - 2 - 3 - 4	0 (0) 5 (6) 26 (31.3) 52 (62.7)	10 (4.9) 7 (3.4) 75 (36.8) 112 (54.9)	0.11
Intracerebral hemorrhage n (%) - Yes	42 (50.6)	64 (31.4)	0.003
Intraventricular hemorrhage n (%)	57 (68.7)	118 (57.8)	0.12
Aneurysm localization n (%) - ACA - ACoA - ICA - MCA - PCoA - BA - VA	4 (4.8) 25 (30.1) 8 (9.6) 20 (24.1) 15 (18.1) 4 (4.8) 7 (8.4)	21 (10.3) 66 (32.4) 16 (7.8) 40 (19.6) 28 (13.7) 16 (7.8) 17 (8.3)	0.62
Median number of aneurysms (range)	1 (1 to 2)	1 (1 to 2)	0.62
Median size of aneurysm (range) mm	6 (4 to 9)	6 (4 to 8)	0.25
Aneurysm circulation n (%) - Anterior - Posterior	72 (86.7) 11 (13.3)	171 (83.8) 33 (16.2)	0.66

mRS = modified Rankin scale, WFNS = World Federation of Neurosurgical Societies, ACA = anterior cerebral artery, ACoA = anterior choroidal artery, ICA = internal carotid artery, MCA = middle cerebral artery, PCoA = posterior communicating artery, BA = basilar artery, VA = vertebral artery

**Table 2:** Treatment characteristics under consideration of shunt-dependency

Characteristic	VP shunt	No shunt required	*p* value
	*n* = 83	*n* = 204	
Aneurysm treatment n (%) - Clipping - Coiling	39 (47) 44 (53)	101 (49.5) 103 (50.5)	0.8
Infarction after aneurysm treatment n (%)	12 (14.5)	22 (10.8)	0.5
Hemorrhage after aneurysm treatment n (%)	5 (6)	20 (9.8)	0.42
CSF drainage management			
- EVD placement n (%) - Mean daily CSF-drainage via EVD in ml (IQR) - LD placement n (%) - Mean daily CSF-drainage via LD in ml (IQR) - Total CSF-drainage day 1–8 in ml (IQR)	73 (88) 159 (97 to 208) 40 (48.2) 108 (84 to 118) 1433 (1009 to 1738)	139 (68.1) 117 (77 to 175) 100 (49) 107 (90 to 118) 1034 (735 to 1490)	< 0.001 0.01 1 0.92 < 0.001
Vasospasm assessment and treatment			
- Clinically suspected vasospasm	32 (38.6)	57 (27.9)	0.1
- TCD-vasospasm > 120 cm/s	40 (55.6)	92 (49.2)	0.44
- TCD-vasospasm > 160 cm/s	20 (27.8)	47 (25.1)	0.78
- Angiographic vasospasm < = 33%	13 (15.7)	20 (9.8)	0.05
- Angiographic vasospasm < = 66%	16 (19.3)	27 (13.2)	
- Angiographic vasospasm > 66%	6 (7.2)	18 (8.8)	
- Endovascular vasospasm treatment	9 (10.8)	15 (7.4)	0.46
Hospital stay - Median length of hospital stay in days (IQR)	30 (24 to 39)	22 (17 to 28)	< 0.001
Discharge destination n (%)			< 0.001
- Home - Rehabilitation - Other hospital	7 (8.5) 67 (81.7) 8 (9.8)	71 (42.3) 86 (51.2) 11 (6.5)	
Infarction at discharge n (%)	27 (32.5)	71 (34.8)	0.82

CSF = cerebrospinal fluid, EVD = external ventricular drainage, LD = lumbar drainage, IQR = interquartile range, TCD = transcranial Doppler sonography

### Necessity of shunt placement

**Fig. 1:**
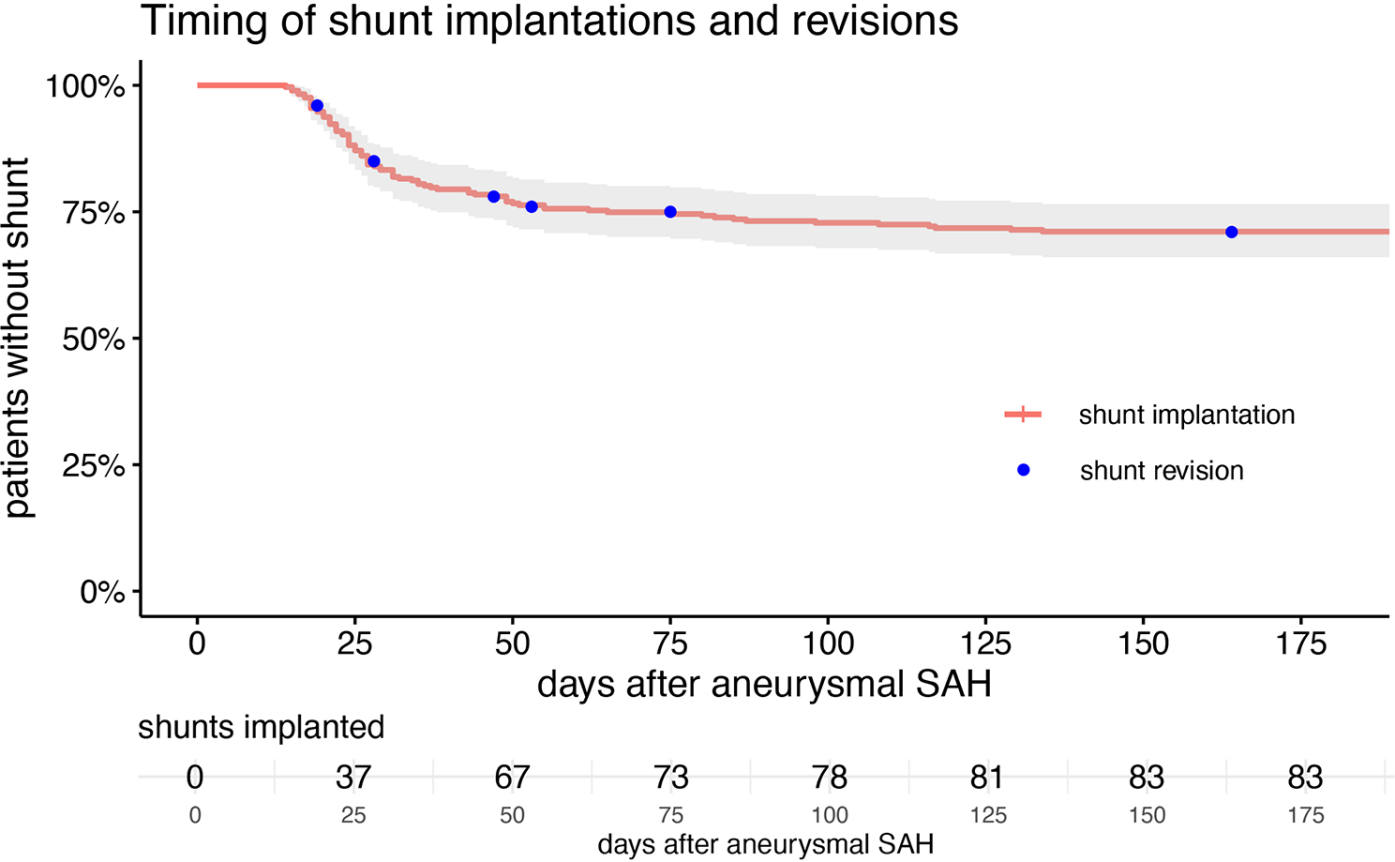
Time frame of shunt insertion and shunt revision in the study population

**Fig. 2:**
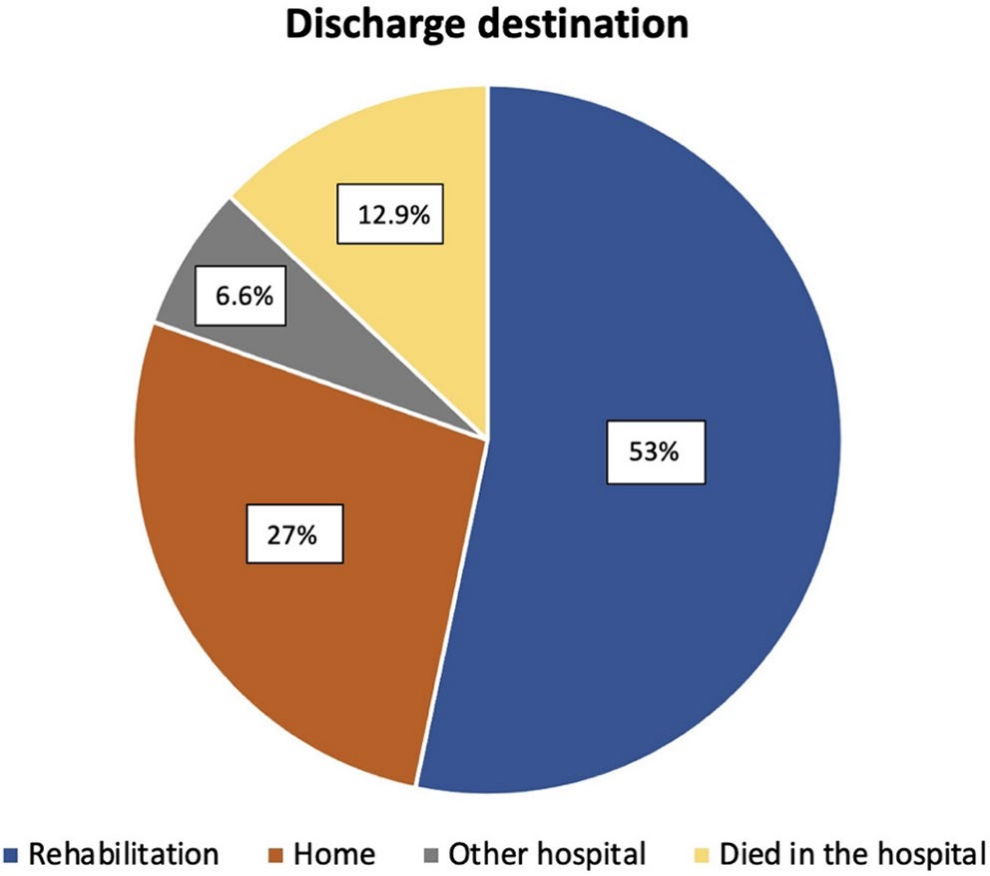
Discharge destinations of the patients with most patients having rehabilitation as their discharge destination

Shunt-dependent hydrocephalus was found in 29% (83/287) of all patients. Median time to shunt insertion was 27 days (IQR 21–45, range 14–134). In 82% (68/83) of cases the shunt was implanted before discharge from initial care and in 18% (15/83) the shunt was placed later after discharge. A shunt-revision surgery was conducted in 7% (6/83) of patients after discharge. The time frame of shunt insertion and revision is depicted in Fig. 1. In 53% (153/287) the discharge destination was a rehabilitation, 27% (78/287) of patients were discharged home, 12.9% (37/287) died in the hospital, one after having a ventriculoperitoneal shunt implanted, and 6.6% (19/287) of the patients were transferred to another hospital (Fig. 2).

In the univariate regression analysis, a shunt-dependancy at 180 days follow-up was associated with higher age (*p* = 0.003), higher WFNS grade (*p* < 0.001), intracerebral hematoma on the initial computed tomography scan (*p* = 0.002), and higher CSF drainage via EVD (*p* < 0.001). A trend to shunt-dependency was found for the presence of intraventricular hemorrhage on the initial imaging (*p* = 0.09) and for symptomatic vasospasm (*p* = 0.08). The multivariate regression analysis revealed higher age (*p* = 0.002), higher CSF drainage via EVD (*p* < 0.001), and symptomatic vasospasm (*p* = 0.01) to be independent factors associated with shunt-dependency at 180 days (Table 3).

**Table 3:** Univariate and multivariate analysis for shunt-dependency

Variables	Univariate analysis			Multivariate analysis		
	OR	95%CI	*p*-value	OR	95%CI	*p*-value
Age, per 10 years	1.38	1.11 to 1.71	0.003	1.45	1.15 to 1.85	0.002
WFNS grade	1.31	1.12 to 1.53	< 0.001	1.16	0.97 to 1.39	0.09
Intracerebral hemorrhage	2.24	1.32 to 3.77	0.002	1.68	0.92 to 3.07	0.09
Intraventricular hemorrhage	1.59	0.93 to 2.74	0.09	1.01	0.54 to 1.85	0.98
Mean EVD per 100 ml	1.93	1.43 to 2.63	< 0.001	1.87	1.34 to 2.61	< 0.001
Symptomatic vasospasm	1.61	0.94 to 2.77	0.08	2.16	1.18 to 3.96	0.01

OR = odds ratio, CI = confidence interval, WFNS = World Federation of Neurosurgical Societies, EVD = external ventricular drainage

### Long-term outcome

**Fig. 3:**
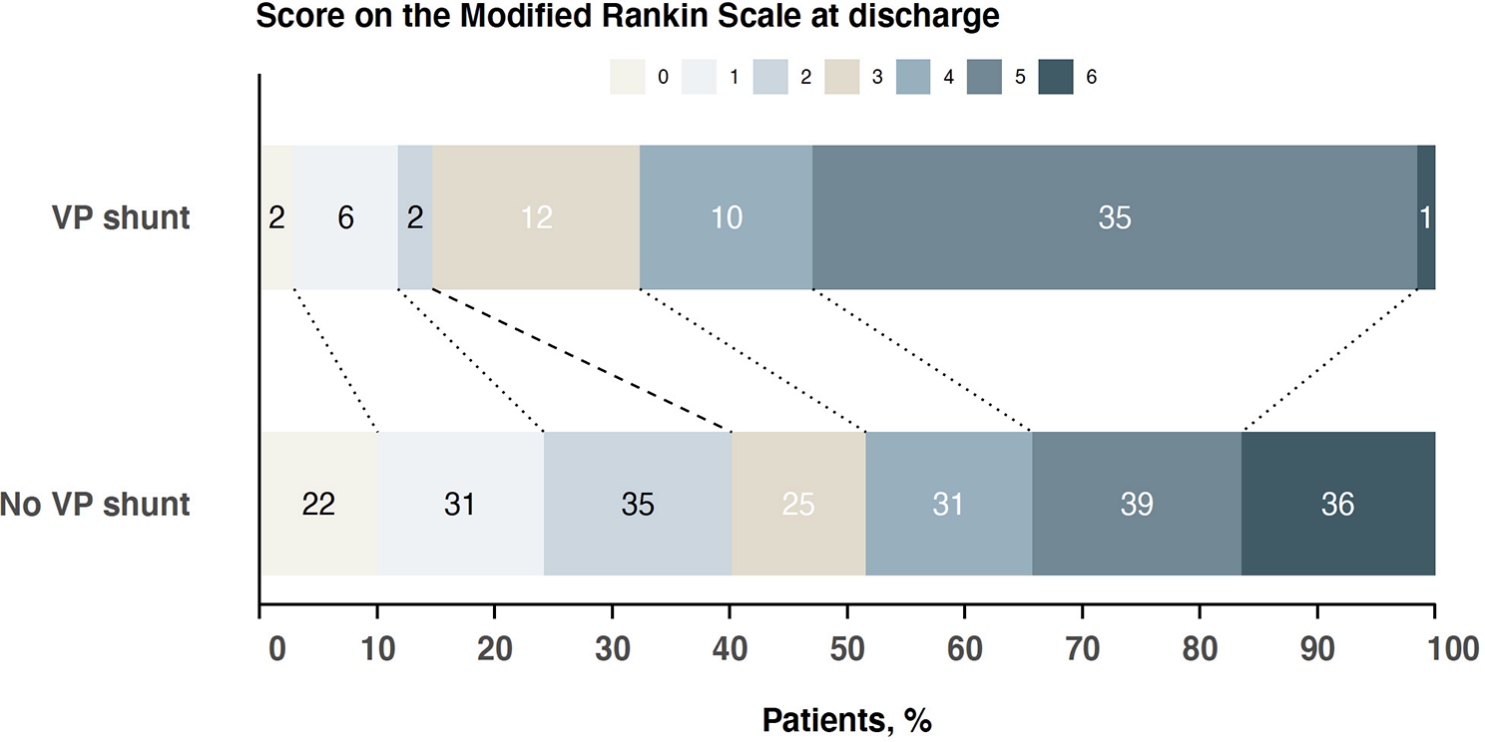
Functional outcome at discharge in the patient group with shunt-dependency compared to those without shunt-dependency. Resembles univariate consideration without adjustment for age, initial hemorrhage severity, and treatment with a lumbar drain

**Fig. 4:**
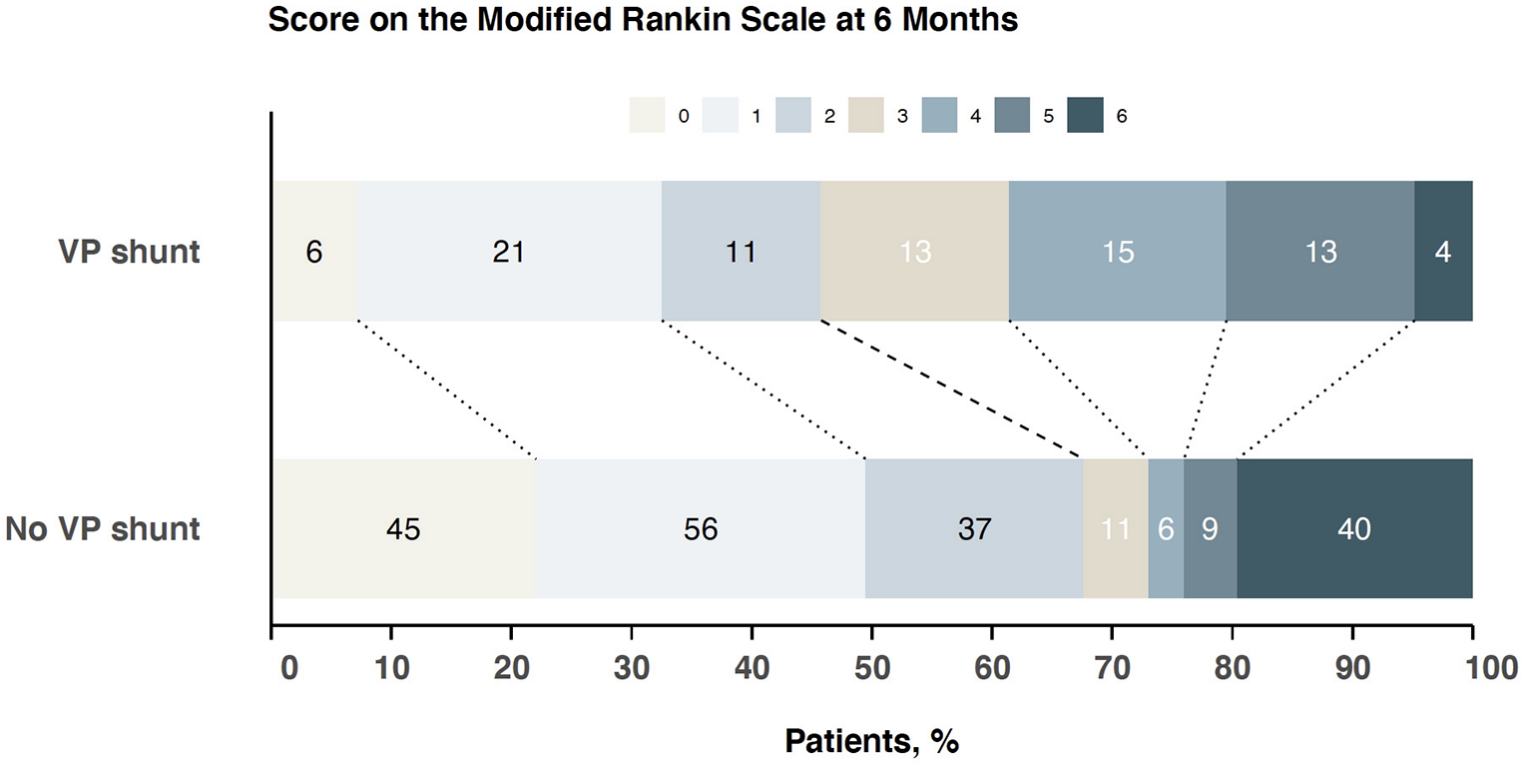
Funtional outcome at 6 months follow-up in the patient group with shunt-dependency compared to those without shunt-dependency. Resembles univariate consideration without adjustment for age, initial hemorrhage severity, and treatment with a lumbar drain

The mean mRS in the study population was 3.4 ± 1.8 (median 4, 95%CI 3 to 4) at discharge and 2.4 ± 2.1 (median 2, 95%CI 1 to 2) at 180 days follow-up. Patients without shunt-dependency had a better functional outcome at discharge (mRS 3.2 ± 2.0), compared to shunt-dependent patients (mRS 3.9 ± 1.5), *p* < 0.001 (Fig. 3). At 180 days, both groups had improved, with again better functional outcome in patients without shunt-dependency (mRS 2.2 ± 2.3 vs. mRS 2.8 ± 1.7, *p* < 0.001), Fig. 4.

In the univariate regression analysis, the following factors were associated with favorable functional outcome: younger age (p = < 0.001), lower WFNS grade (*p* < 0.001), absence of intraventricular hemorrhage (*p* < 0.0001) or intracerebral hematoma (*p* < 0.0001) on the initial computed tomography, CSF drainage via LD (*p* = 0.01), and absence of shunt (*p* < 0.001). In the multivariate analysis younger age (*p* < 0.001), lower WFNS grade (*p* < 0.0001), absence of intracerebral hemorrhage and treatment with a lumbar drain remained relevant factors, while shunt requirement lost its significant relationship with outcome (*p* = 0.26, Table 4).

**Table 4:** Univariate and multivariate analysis for functional outcome at 6 months follow-up

Variables	Univariate analysis			Multivariate analysis		
	OR	95%CI	*p*-value	OR	95%CI	*p*-value
Age, per 10 years	1.57	1.27 to 1.94	< 0.001	1.55	1.22 to 1.97	< 0.001
WFNS grade	1.71	1.45 to 1.99	< 0.001	1.57	1.31 to 1.87	< 0.001
Intracerebral hemorrhage	3.55	2.14 to 5.88	< 0.001	1.98	1.10 to 3.56	0.02
Intraventricular hemorrhage	2.90	1.71 to 4.91	< 0.001	1.81	0.99 to 3.32	0.05
Treatment with lumbar drain	1.88	1.13 to 3.11	0.01	1.88	1.05 to 3.38	0.03
Shunt placement anytime	2.47	1.46 to 4.17	< 0.001	1.42	0.77 to 2.60	0.26

OR = odds ratio, CI = confidence interval, WFNS = World Federation of Neurosurgical Societies

## Discussion

In this secondary analysis of the EARLYDRAIN trial, we were able to confirm age, hemorrhage severity and drainage volume via EVD but not LD age as relevant predictors for shunt dependency in chronic hydrocephalus after aSAH. In multivariate analysis, shunt dependency was found not to be related to worse long-term outcome, measured by the mRS after 6 months. In contrast to most available literature dealing with retrospective cohort analyses, the main asset of the present work is the availability of prospectively recorded data. Chronic hydrocephalus requiring permanent cerebrospinal fluid diversion occurs in 9–36% of aSAH patients [4]. Higher age, higher WFNS grade, presence of intraventricular hemorrhage, angiographic vasospasm, ruptured aneurysm of the posterior circulation, acute hydrocephalus, and the need for decompressive hemicraniectomy were reported in the literature as independent predictors of shunt-dependency after aSAH [12–14]. Almost all risk factors for shunt-dependent hydrocephalus are non-modifiable parameters, which impedes a proactive risk reduction for developing a shunt-dependent hydrocephalus. Consequently, these parameters can be used for identification of patients at risk for developing shunt-dependent hydrocephalus but not for preventing shunt-dependency. Age, aneurysm size, and the total CSF drainage volume were independent factors associated with shunt-dependency in our study cohort, which is partially in line with the current literature [15–17]. The CSF drainage volume represents at least in the theory a potentially modifiable parameter. However, higher CSF drainage volumes are rather regarded as a sign of a CSF circulation dysfunction than as a factor that will result in the prevention of hydrocephalus by actively reducing the CSF drainage volumes. The presence of a CSF circulation dysfunction causes an increase in intracranial pressure which in turn results into higher CSF drainage volumes. Higher CSF drainage via EVD was associated with increased shunt-dependency, while, interestingly, the use and rate of lumbar drainage was not. This points again to the fact that the way of CSF drainage matters in patients with aSAH. It seems like the lumbar drainage is contributing to a better restoration of the CSF circulation compared to EVD because its following the physiological CSF flow direction.

Another frequently evaluated factor in this context is the weaning form of the EVD (rapid vs. gradual), that was recently addressed in a meta-analysis demonstrating no impact of the wean strategy on the shunt insertion rate [18, 19]. The upcoming results of the DRAIN trial are likely to add further evidence to this subject [20]. Multivariate analyses on our study revealed an independent association with shunt-dependency at 180 days follow up, which was an interesting finding of this post-hoc analysis. Symptomatic vasospasm was defined as clinically suspected vasospasm, that per definition can be only stated in awake patients, in whom a clinical assessment can be performed.

Shunt-dependency is deemed to have a negative influence on outcome due to persisting symptoms despite of the shunt placement on the one side as well as due to associated complications with shunting such as infections or dysfunctions [14]. Shunt-dependent patients had lower return to functional independence rates and lower return-to-work rates [14]. The shunted cohort tended toward worse outcome because they had a higher grade of hemorrhage [14]. While several studies demonstrated a negative impact of shunt-dependent hydrocephalus on short-term outcome of aSAH patients, the role of shunt-dependency on the long-term functional outcome after aSAH has not been determined yet. Paisan et al. reported an association of shunt-dependency with unfavorable outcome (mRS 3–6) with a 44% rate of functional dependency or death at last follow-up [4]. In the current post-hoc analysis of the EARLYDRAIN data, at first glance, shunt-dependency led to worse functional outcome at discharge and after 180 days when considered in isolation. However, shunt-dependency showed no significant association with the long-term outcome at 180 days follow-up when adjusted for initial hemorrhage severity. This finding initiates a paradigm shift regarding shunt-dependency following aSAH, pointing out that shunt-dependent hydrocephalus per se is not an independent risk factor for unfavorable long-term outcome after aSAH. A reversal of hydrocephalus-related symptoms after shunt insertion is the most obvious explanation for this finding. Additionally, further clinical improvement through rehabilitation (from mean mRS at discharge 3.9 to 2.7 at 180 days) may also have contributed to these results, since 83% of shunt-dependent patients were discharged to rehabilitation. Hence, the clinical focus should rather be on factors associated with favorable long-term outcome than on factors associated with shunt-dependency after aSAH. Longer time interval from aneurysm rupture to shunt placement, lower rates of shunt infections, and endovascular aneurysm occlusion have been previously shown to be associated with favorable outcome in shunt-dependent patients after aSAH [4]. The choice of aneurysm treatment modality had no impact on shunt-dependency in our study. This was also confirmed by Zaidi et al. who found no difference in the shunt-dependency rate between patients treated with clipping and those treated with coiling [14]. In a recently published article early shunt placement (within 21 days after ictus) showed no difference in the complication rate and in overall mortality, compared to late shunt placement more than 21 days after ictus [21]. This was in line with the findings of our study.

### Limitations of the study

The functional outcome of patients with aSAH is determined by multiple factors. Some of these may have been missed in this post-hoc analysis; hence, we cannot exclude an impact on the findings of our study. We do not have knowledge of drainage weaning strategies applied from local caregivers. As shunt-dependency was not the primary objective of the EARLYDRAIN trial, no detailed data are available concerning complications like shunt infections. Lastly, although EARLYDRAIN did enroll patients of all severity grades, the analysis lacks power when distinct subgroups are in consideration. A larger database with more poor-grade patients and higher prevalence of shunt requirement may retain shunt-dependency as negative outcome factor when investigated multivariate. The same may apply for analyzing subsets of patients, like only those surviving initial care, or only patients presenting with initial hydrocephalus and requiring and EVD in primary care. Ultimately, this will contribute to the high variance of shunt necessity after aSAH reported in literature.

## Conclusions

Shunt-dependency did not show an effect on long-outcome in aSAH patients after rehabilitation. Clinical improvement after shunt insertion is a likely explanation why chronic hydrocephalus per se does not represent an independent predictor of long-term outcome. The findings of this post-hoc analysis suggest that shunt-dependent hydrocephalus does not have to be feared as an outcome-limiting complication of aSAH.

## Data Availability

No datasets were generated or analysed during the current study.
